# Domain randomization-enhanced deep learning models for bird detection

**DOI:** 10.1038/s41598-020-80101-x

**Published:** 2021-01-12

**Authors:** Xin Mao, Jun Kang Chow, Pin Siang Tan, Kuan-fu Liu, Jimmy Wu, Zhaoyu Su, Ye Hur Cheong, Ghee Leng Ooi, Chun Chiu Pang, Yu-Hsing Wang

**Affiliations:** 1 Department of Civil and Environmental Engineering, The Hong Kong University of Science and Technology, Hong Kong, SAR China; 2 Division of Integrative Systems and Design, The Hong Kong University of Science and Technology, Hong Kong, SAR China; 3 School of Biological Sciences, The University of Hong Kong, Hong Kong, SAR China; 4 Hong Kong Bird Watching Society, Hong Kong, SAR China

**Keywords:** Behavioural ecology, Animal behaviour

## Abstract

Automatic bird detection in ornithological analyses is limited by the accuracy of existing models, due to the lack of training data and the difficulties in extracting the fine-grained features required to distinguish bird species. Here we apply the domain randomization strategy to enhance the accuracy of the deep learning models in bird detection. Trained with virtual birds of sufficient variations in different environments, the model tends to focus on the fine-grained features of birds and achieves higher accuracies. Based on the 100 terabytes of 2-month continuous monitoring data of egrets, our results cover the findings using conventional manual observations, e.g., vertical stratification of egrets according to body size, and also open up opportunities of long-term bird surveys requiring intensive monitoring that is impractical using conventional methods, e.g., the weather influences on egrets, and the relationship of the migration schedules between the great egrets and little egrets.

## Introduction

Bird detection, including bird localization, classification, counting and density estimation, is crucial for different applications of ornithological studies, such as investigating the stopover pattern of birds^1–3^; exploring the long-term influence of climatic change on the arrival behavior of migrant birds^4^ and identifying the habitat selection of different bird species^5,6^. However, such research remains challenging as manual observation by experts, which is time-consuming and labor-intensive, is the primary method for bird detection. Consequently, the collected data are often fragmentary and of low-frequency and coarse-spatial-resolution^7^, resulting in a lack of training data. Although radar scanning^8,9^ and traditional computer vision techniques^10–12^ have been applied to automate bird detection, these techniques are highly influenced by the environment (e.g., trees and buildings)^13,14^ and ineffective in extracting fine-grained features that are needed by experts to distinguish bird species^15^. The emergence of deep learning^16–24^, specifically models used for object detection^25–28^, has provided opportunities to enhance the efficiency and accuracy of bird detection algorithms^14,29–31^. For instance, deep learning-based semantic segmentation models are used to detect birds and other objects (sky, cloud, forest and wind turbine) from images taken at wind farms^29–31^; different deep learning-based object detection models (e.g., Faster R-CNN^32^, YOLO^33^ and RetinaNet^34^) are evaluated for detecting birds from aerial photographs collected by an unmanned aerial vehicle^14^. However, these studies focus on distinguishing birds from other objects, instead as classifying different bird species. Some studies focus on bird species classification, e.g., LeNet, a simple deep learning-based classifier was used for classifying hawk and crow from images^35^. Other training strategies have also been developed to extract fine-grained features for deep learning-based image classification^15,36–40^. Nevertheless, most of these strategies are inappropriate for the bird detection in this project. For instance, the feature maps produced by bilinear convolutional neural networks that generate second order bilinear features to describe two-factor variation (e.g., “style” and “content”)^36,37^ are extremely high-dimensional and require excessive computational memory. Next, the limited amount of labeled bird detection data may cause overfitting and restricts the applicability of the domain-specific transfer learning^39,40^. In addition, objects of low spatial resolution might reduce the capacity^41^ of the weak supervision^15,34^ in extracting the fine-grained features.

**Figure 1:**
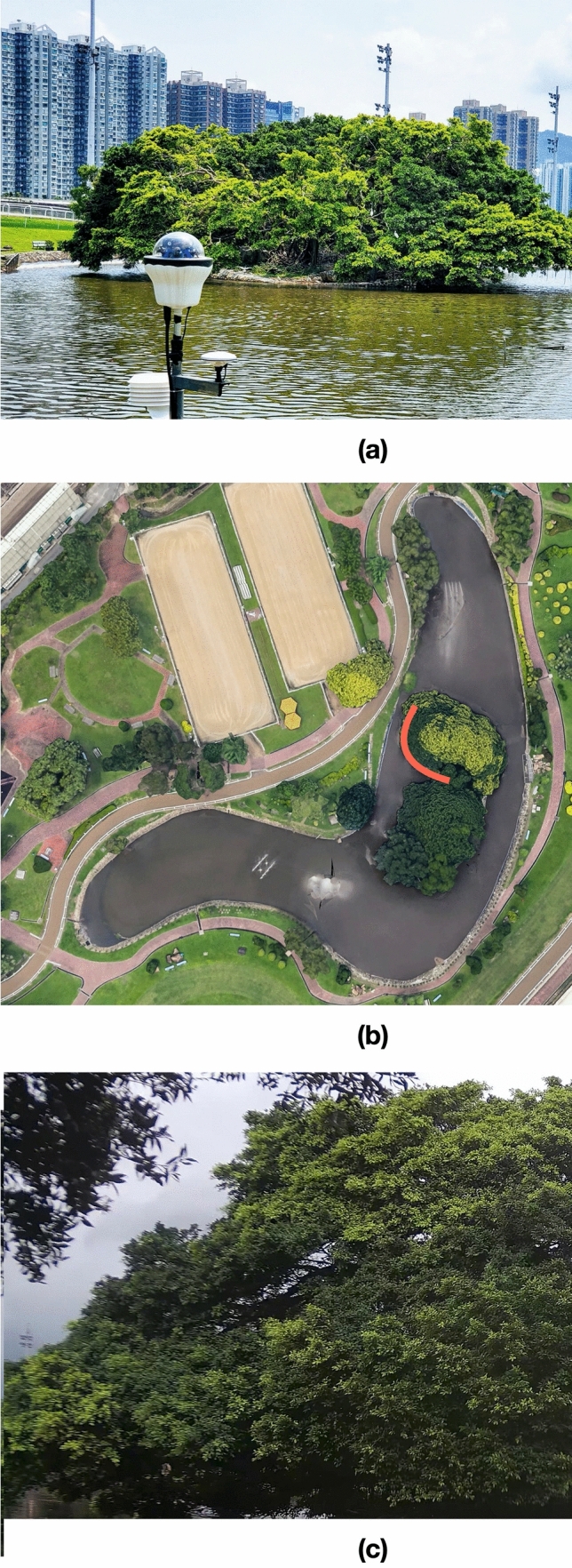
Penfold Park, the study site: (**a**) Green AI camera used to automatically record bird videos at this study site; (**b**) aerial view of the study site. The orange region represents the viewpoint of Green AI Camera; and (**c**) trees at the centre of the pond, which is the background image recorded by Green AI Camera.

**Figure 2:**
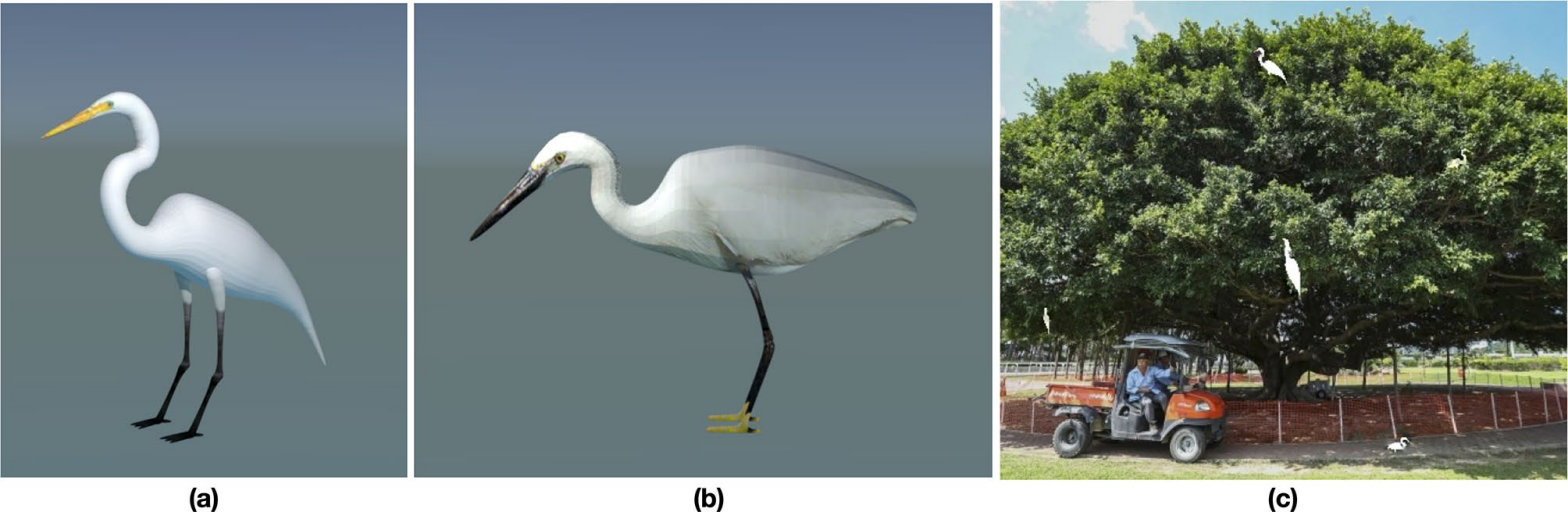
Application of domain randomization to enhance the accuracy of the detection models. Virtual great egret (**a**) and little egret (**b**) were created with the open-source 3D graphics toolset Blender. Synthetic image (**c**) was generated by merging virtual 3D models and 2D background images. When merging, we applied a large variety of the prominent features, such as body size and pose, at different camera viewpoints and locations at the images, in order to force the models to focus on the fine-grained bird features, which are essential features used by experts for bird identification.

**Figure 3:**
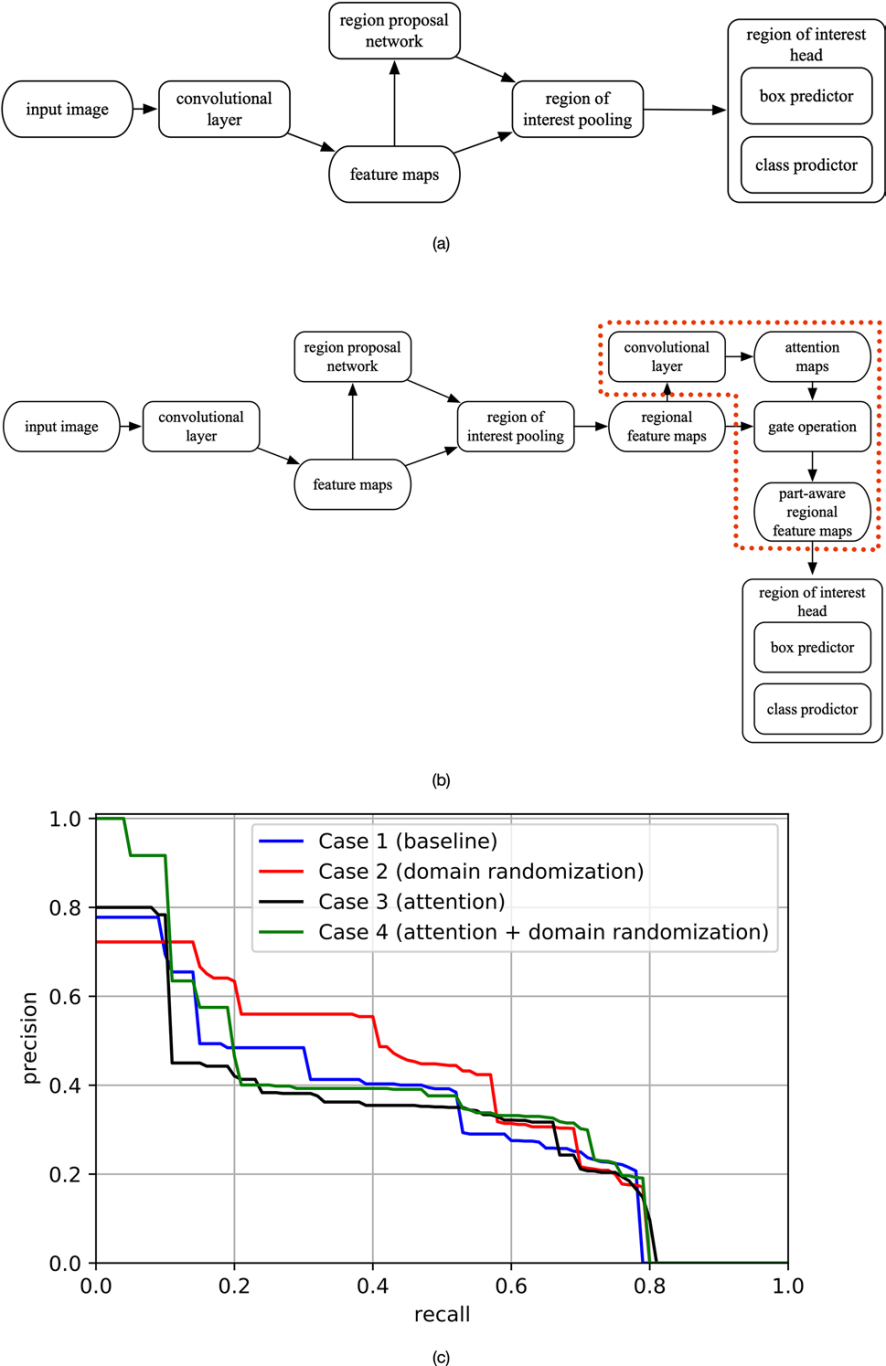
Object detection model used in this study for bird detection and the performance results of the related experiments: (**a**) Faster R-CNN, which is the state-of-the-art model in most object detection tasks. In this study, we selected ResNet-50 as the backbone for feature extraction (represented by the “convolutional layer” stage of the figure). We also attached Feature Pyramid Network to Faster R-CNN to render effective fusion of multiscale information; (**b**) Object detection models trained with the attention mechanism (represented by the dotted region), which was used for the performance comparison with our proposed domain randomization-enhanced deep learning model; and (**c**) precision-recall curve (at IoU = 0.5) for Case 1 (baseline), Case 2 (domain randomization), Case 3 (attention) and Case 4 (attention + domain randomization).

Thus, alternatives are urgently needed to tackle the aforementioned challenges. We first designed and installed a tailor-made Green AI Camera (see Fig. 1a and “Methods”) at the study site, i.e., Penfold Park, Hong Kong, China (see Fig. 1b), to automate the data collection steps of the bird videos. In addition to saving manpower for manual bird detection, months-long data that were automatically acquired by this advanced vision sensing device enabled comprehensive analyses on bird behavior. Next, we adopted domain randomization to enhance the accuracy of deep learning models for bird detection from the recorded video data, which is the main focus of this study. Domain randomization, which makes use of virtual objects and/or environments to augment the data variability for model training, are found to be effective in building models that are generalized to different complex real-world circumstances^42^. For example, domain randomization with synthetic data helps to train robotic manipulation for grasping specific objects^43^ and for learning collision avoidance^44^ and gets promising results. Inspired by these studies, we generated synthetic data by creating virtual birds (great and little egrets shown in Fig. 2) in different environments so as to further augment the data size, and at the same time, to ensure sufficient variations for rendering the fine-grained bird features, i.e., neck and head, that are used by experts to distinguish bird species. Then, we pretrained the Faster R-CNN^32^ (a deep learning detection model; see Fig. 3a) with the synthetic data, i.e., virtual birds with different real-world backgrounds, followed by fine-tuning the model with real data collected at the test site. Based on the detection results of the continuously monitored data, we conducted analyses to study the bird behaviour, that were practically difficult in the past due to expensive data collection and limited labeled data. Our results not only provide more evidence to support previous studies (e.g., nest site selection of the great egrets and little egrets), but also reveal interesting findings (e.g., weather influences and daily schedule of egrets), suggesting the potential applications of our proposed innovation for better habitat management, conservation policies and protection measures.

## Results

### Performance evaluation of the domain randomization-enhanced deep learning model

**Figure 4:**
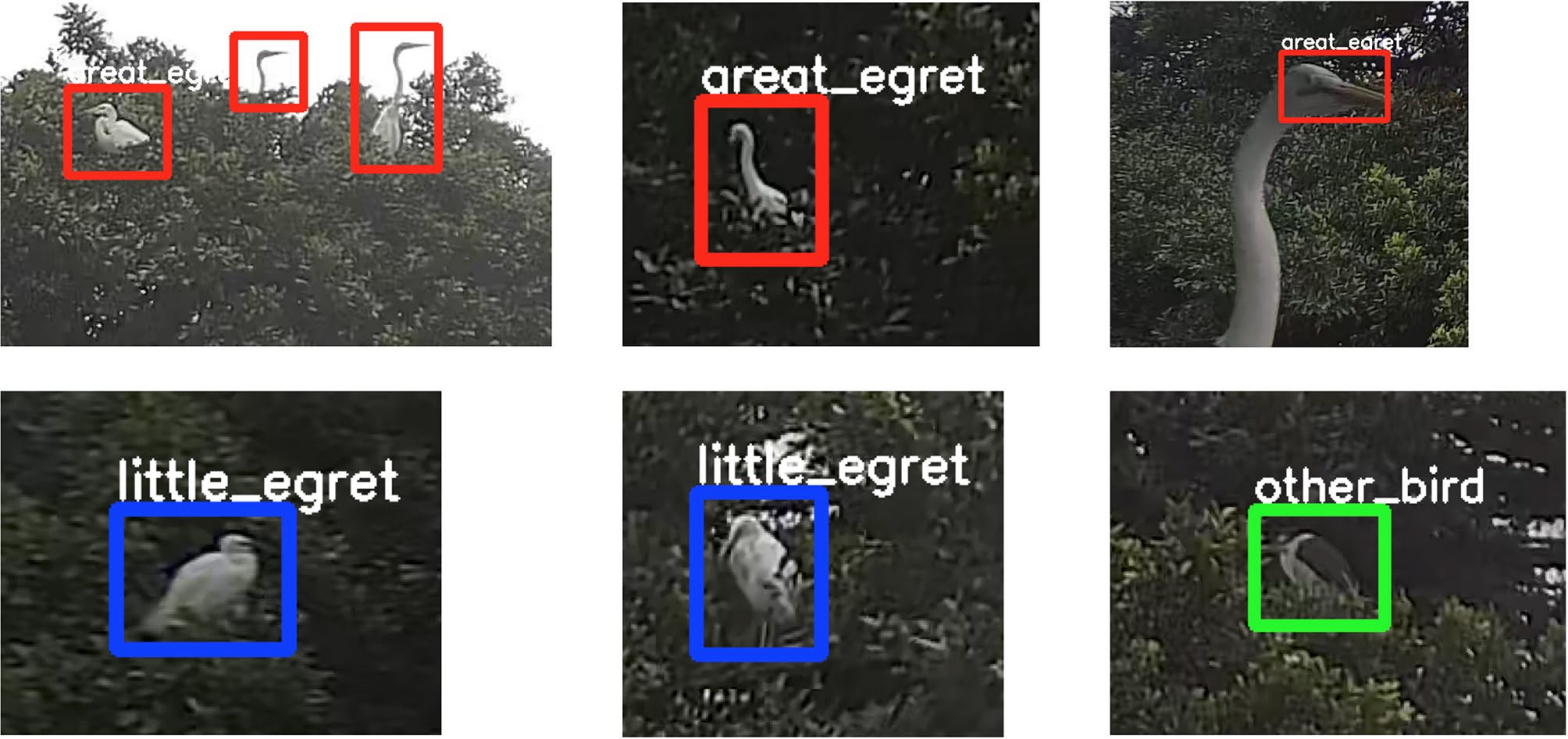
Example of bird detection result. The blue, orange and green bounding boxes are used for the predictions of the great egrets, little egrets and other birds.

We selected Faster R-CNN^32^ to detect different bird species from real images, including great egrets, little egrets and other birds (mainly black-crowned night herons). ResNet-50^45^ was used as the backbone for feature extraction, and Feature pyramid network (FPN)^46^ was applied to efficiently extract multiscale features. The real-world data was collected with the Green AI Camera (see “Methods”). The labeled data were split into 900, 100 and 110 images as the training, validation and testing sets (see Methods). With domain randomization, we generated 1000 synthetic images for model pretraining, then fine-tuned the model with the 900 real images. Figure 4 depicts an example of the detection result, and the analysed video is provided in the Supplementary Video. The domain randomization-enhanced model is capable of distinguishing and localizing bird species with high prediction scores under different backgrounds (e.g., clear sky, and partially covered by leaves and branches), achieving a mean average precision (mAP, at the intersection over union of 0.5) of 87.65% (see “Methods”).

**Figure 5:**
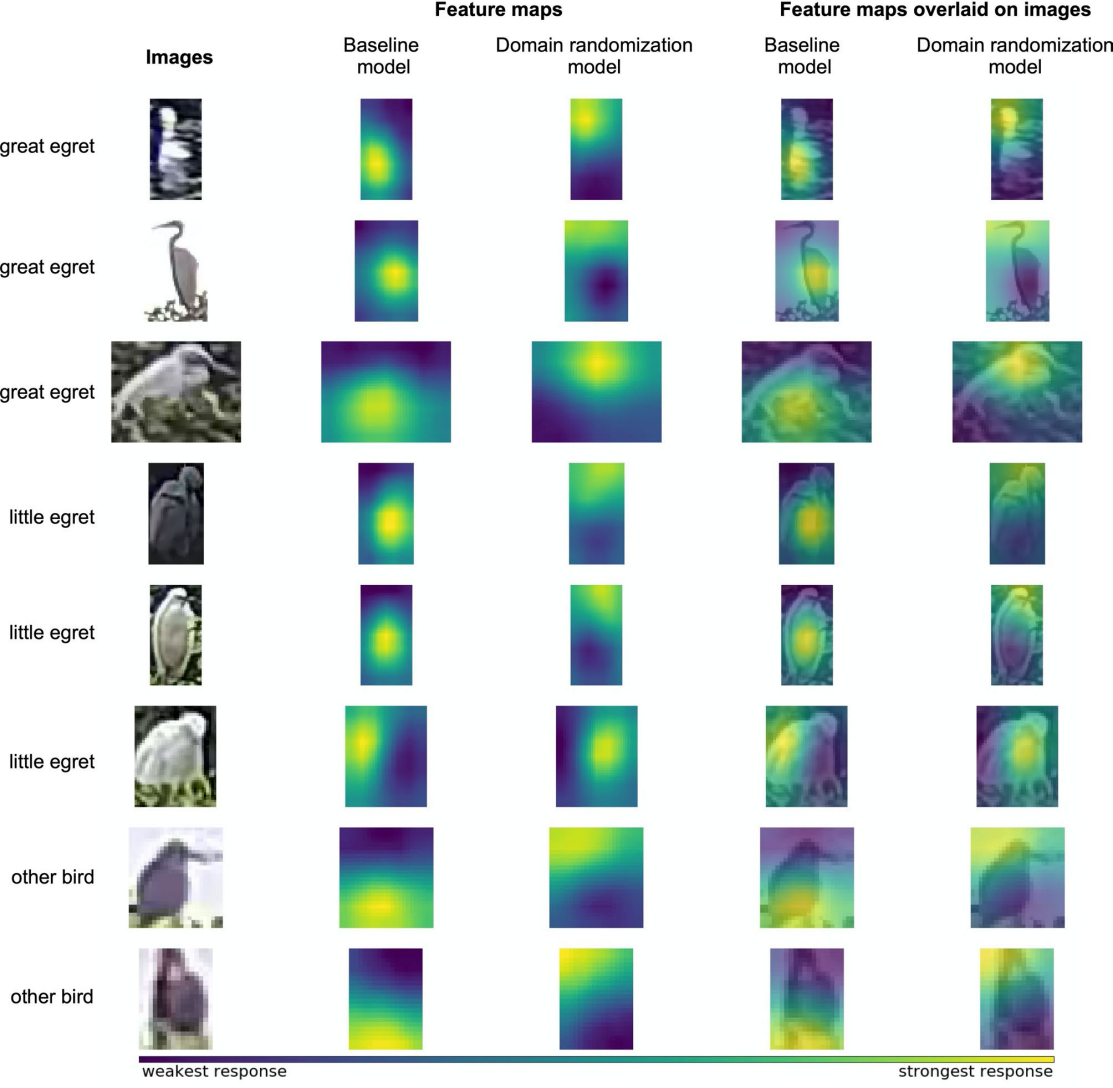
Visualization of the perception made by detection models for distinguishing bird species. We compared our proposed method with the same architecture that is trained with real images only (known as the baseline model). For examples of different bird species (first column), the feature maps (response of the model) of the baseline model and domain randomization-enhanced model are shown in second and third column, respectively. The viridis colormap is used to illustrate the intensity of the response, where yellow represents the strongest response. The feature maps are overlaid on the bird images to depict the features used by the baseline model (forth column) and the domain randomization-enhanced model (fifth column) for bird detection. We realize that model pretraining with the synthetic images forces the model to focus on the fine-grained features, such as neck shape and beak color, which are the crucial features used by experts for bird identification.

We next examined the advantages of domain randomization on augmenting the detection accuracy. Figure 5 depicts the perception achieved by the models in distinguishing bird species, based on the feature maps computed at the last layer of the ResNet-50 backbone, together with the baseline model (Faster R-CNN trained with real images only) for comparison. We observe that the domain randomization-enhanced model focuses on the subtle features of the neck and head, which are the essential fine-grained features used by the experts to distinguish bird species. On the other hand, the baseline model tends to identify bird species from the color and textural features of the body, which may not be the optimal criteria. It should be noted that the bird size, which is one of the features used by human experts, is not considered by the deep learning models as the observed size could change over the distance from camera, and the depth information is unavailable from the images.

We further examined the effectiveness of domain randomization by performing quantitative evaluation. In addition to the baseline model (Faster R-CNN trained with real images only), the weakly supervised method based on attention mechanism^15^ (Fig. 3b), which locates the “parts” of birds and then extract the fine-grained features, was also used as a training strategy for comparison. We used a small subset of the real images for the comparison to highlight the strength of the domain randomization under a limited amount of labeled data, which is a common challenge in most of the fine-grained visual recognition tasks^39^. The training set was made up of 60 real images (136 great egrets, 43 little egrets and six other birds) and 1000 synthetic images; the validation set comprised 40 real images (85 great egrets, 12 little egrets and eight other birds) and the test set comprised 40 real images (124 great egrets, 21 little egrets and 10 other birds), respectively. Summarizing, we considered four cases in our comparison:

1. Case 1 (baseline): Faster R-CNN, trained with the real images only.
2. Case 2 (domain randomization): Faster R-CNN, pretrained with the synthetic images, and fine-tuned with the real images.
3. Case 3 (attention): Faster R-CNN with attention mechanism, trained with real images only.
4. Case 4 (attention + domain randomization): Faster R-CNN with attention mechanism, pretrained with the synthetic images, and fine-tuned with the real images.

We used mAP (IoU = 0.5) to evaluate the performances of all the four cases based on the test set (see “Methods”). The reported mAPs (± standard deviation) of the four cases are 0.342 ± 0.017, 0.397 ± 0.017, 0.329 ± 0.031 and 0.385 ± 0.036. For referencing, the precision-recall curve (at IoU = 0.5) for all four cases is also presented (see Fig. 3c), which indicates information about the trade-off between precision and recall values at different sensitivity threshold. The domain randomization-enhanced model (Case 2) achieves the most balanced result, with better precision scores at a wide range of recall scores between 0.11 and 0.58. Consistent with the previous analysis, Case 2 outperforms the baseline model (Case 1), asserting the effectiveness of the synthetic data with sufficient variations in enabling the models to focus on the fine-grained features of the birds. Although the attention mechanism-based model has state-of-the-art performance in some fine-grained visual categorization tasks^15^, overall the model performance is unstable in our study, represented by the relatively low mAP and high standard deviation of Cases 3 and 4. We believe that the failure in gaining advantage of the attention mechanism is due to the restricted resolution of the regional feature maps of the birds (output of the “region of interest pooling” stage shown in Fig. 3b)^39,41^, as the original dimension of bird is small (i.e., about 0.095% of the image size, see “Methods”).

### Analyses of the egret behaviour

Our analyses focus on the great egrets and little egrets because they are the dominant species at the study site, according to the on-site monitoring and the observations during data annotation. The following analyses are based on the detection results of the videos recorded within the periods 2019-09-23–2019–11-26, which is about 2 months, and the data size is about 100 terabytes.

**Figure 6:**
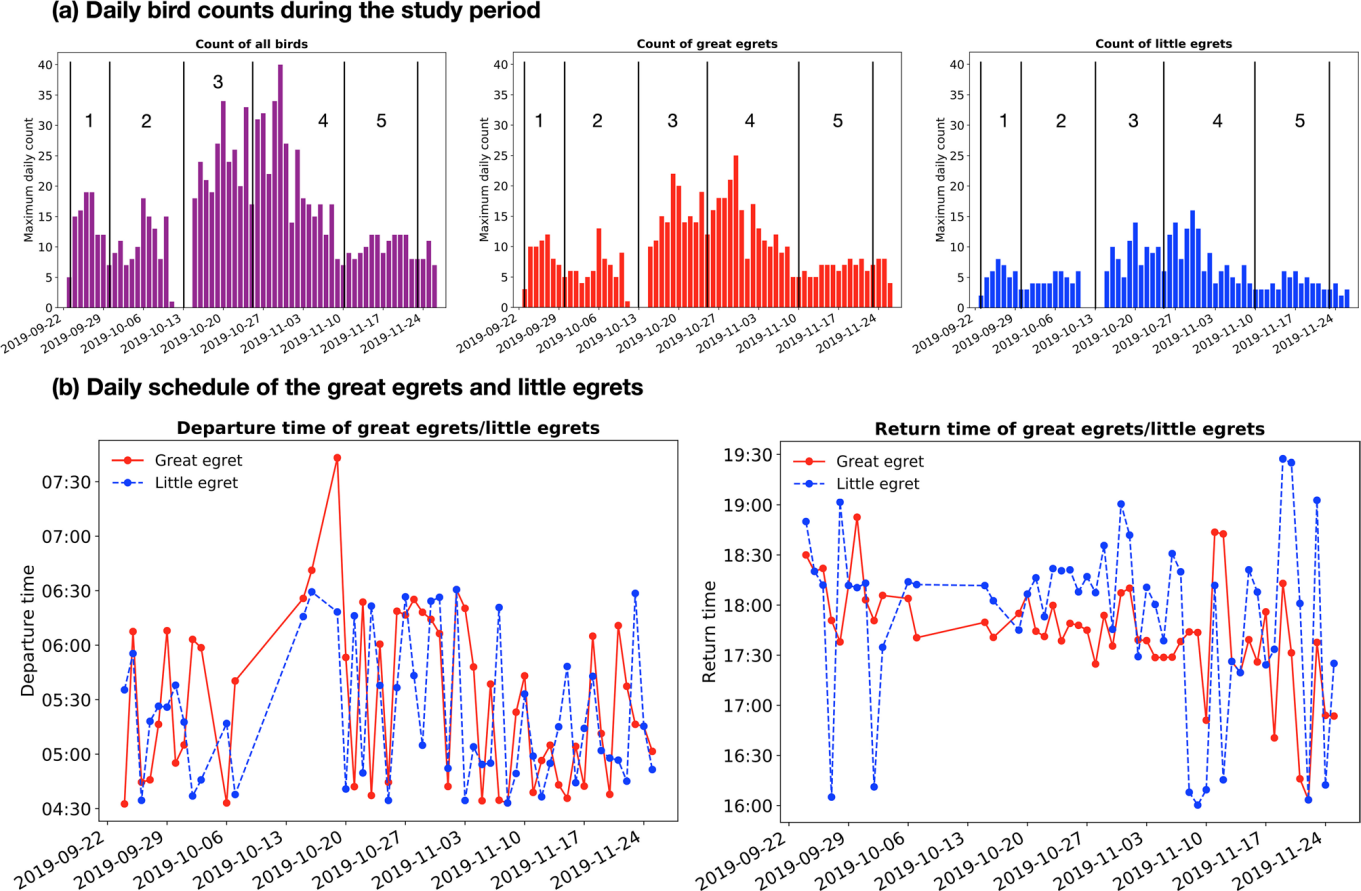
Analyses of the egret behaviour from the temporal perspective: (**a**) Daily bird counts during the study period. The bar graphs show the counts of all birds, great egrets and little egrets. We observe the bird counts increase from 2019-09-23 to 2019-09-27 and then decreases from 2019-09-27 to 2019-09-30. Such a trend repetitively occurs at the periods 2019-09-30–2019-10-13, 2019-10-13–2019-10-25, 2019-10-25–2019-11-10 and 2019-11-10–2019-11-23. All these sub-periods are separated with black lines in the figure. We also realize a surge in bird counts between the end of October and the beginning of November, which is believed to be attributed to the presence of migratory population; (**b**) Daily schedule of the greats and little egrets. The departure and return time of the egrets are estimated from the peak hourly counts in the morning and evening, respectively.

Figure 6a presents the daily counts of all birds, great egrets and little egrets during the study period based on the detection results. The daily counts (vertical axis of Fig. 6a) are inferred from the maximum number of birds detected in a single frame per day and presumed as the number of birds staying at the study site. The great egret numbers increase and then decrease during the period 2019-09-23–2019-09-30. This trend repetitively occurs at the periods 2019-09-30–2019-10-13, 2019-10-13–2019-10-25, 2019-10-25–2019-11-10 and 2019-11-10–2019-11-23. This observation is consistent with the weekly count of the great egrets in Hong Kong between 1958 and 1998, as documented by the Hong Kong Bird Watching Society^47^. We also notice a surge of the great egret counts between the end of October and the beginning of November, which can be explained by the influx of the migratory population during this period^47^. For the little egrets, our analysis shows that similar trend of counts as the great egrets exists, with the Pearson correlation coefficient of 0.89, suggesting the highly-correlated migratory activities between the two species. While supporting the existence of migratory population of the little egrets^47,48^, this finding also motivates us to conduct more studies about the migratory behaviour of and the interaction between egrets.

We scaled down the monitoring duration to observe the daily schedule of egrets. For each individual day, we calculated the backward 1-h moving average of the bird counts for each time point, then presumed the time point with the maximum average value in the morning and evening as the departure and return time of the egrets. The departure times of the great egrets and little egrets are similar during the study period (from 04:33 to 07:31, see Fig. 6b), supported by the hypothesis testing HT1 (p-value = 0.296, which is not statistically significant to reject that the departure times are similar; see “Methods”). On the other hand, the little egrets return later than the great egrets on most of the days (from 16:03 to 19:10, see Fig. 6b), with the reported p-value = 0.098 (marginally significant to reject that the little egrets return earlier than or at the same time as the great egrets, in HT2). We believe that the daily schedule of the egrets are highly related to the prey availability in different foraging habitat preferences (e.g., at Hong Kong, little egrets mostly forage at commercial fishponds and great egrets forage at mudflats^49,50^).

**Figure 7:**
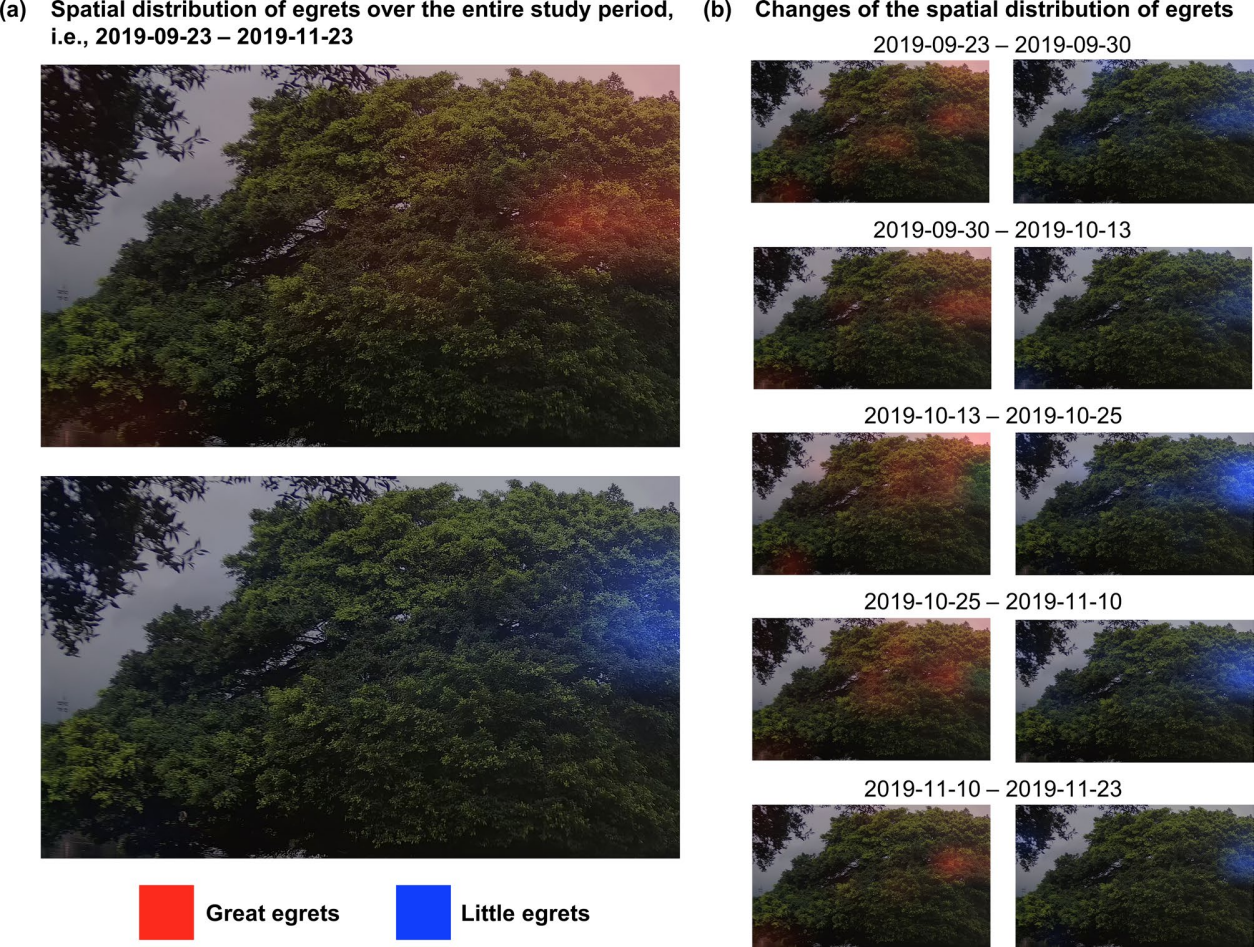
Spatial distribution of the great egrets (red) and little egret (blue) at the study over (**a**) the entire study period; and (**b**) the testing periods split based on the results observed at Fig. 6a to observe the associated changes. The intensity of hotspot reflects the bird counts at a region, which could be used as an indicator to study the nest site selection and habitat preference of different bird species.

In addition to the temporal perspective, we analysed the spatial distribution of egrets at the study site via heatmap visualization (see “Methods”). We observe some horizontal overlap for the great egrets and little egrets in the middle right regions of the trees (see Fig. 7a). However, in terms of elevation, the coverage of the great egrets is wider, i.e., from the middle to the top of trees (Fig. 7a), whereas the little egrets mainly stay around the middle region of the trees (Fig. 7a). This pattern of vertical stratification, to a certain extent, is consistent with the observations of other studies^51–53^, where birds align themselves vertically according to the actual body size (not the body size represented in images, which is influenced by the distance to the camera). Furthermore, we split the testing periods according to the trends observed in the bird counts (Fig. 6a) to identify the spatial change of the egret hotspots (Fig. 7b). For great egrets, the hotspot near the middle right region of the trees remains relatively constant in size and intensity, whereas the hotspots at the top of the trees (vulnerable to wind) shrink and grow over time, and similar changes are observed for the little egrets. Based on the aerial view (Fig. 1b), we observe that the hotspots (at the middle right region of Fig. 7b) are sheltered from wind/gales in the north/south direction, which renders this location a favorable habitat for egrets to inhabiting, breeding and hatching.

We attempted to probe the relationship between weather factors and bird activities (reflected from the bird counts) by building a multivariable linear regression model. The weather data were collected from the Hong Kong Observatory Open Data^54^. We used the natural logarithm of the total bird counts to reflect its proportional change over the study period. We detrended the data based on the hypothesis testing HT3 (p-value = 0.025, which is statistically significant to reject that the time has no influence on the regression; see “Methods”). Table 1 summarizes the multivariable linear regression analysis. We realized that the influence of the combined weather factors is highly statistically significant (p-value of the F-test = 0.002) and undertook further analysis on each factor. Total Bright Sunshine (p-value = 0.031) is statistically significant in affecting bird activities, consistent with previous studies reporting that sunlight influences migration, foraging and other activities of birds^55–57^ Prevailing Wind Direction (EW, p-value = 0.011) is also statistically significant, suggesting that wind has negative effect on nestlings survival^51^. Although several studies suggested that temperature and humidity might play crucial roles and affect the bird behaviour^58,59^, e.g., varying air temperature and humidity might influence water temperature, that possible alter the activity levels of forage fishes, which in turn affects the foraging efficiency of egrets^60^, our results in Table 1 shows that the Daily Mean Temperature (p-value = 0.080) and Mean Relative Humidity (p-value = 0.089) are just marginally significant. While supporting the influence of some weather factors in affecting the bird behaviours, more rigorous analysis (e.g., collecting in situ measurements using a local weather station) is required to provide evidence to validate the hypothesis.

**Table 1:** Multivariable linear regression analysis.

Overall model fit		
Number of observations	62	
R^2^	0.434	
p-value (F-test)	0.002	
**Parameter estimates**		
Dependent variable	ln (count of all birds)	
**Independent variable**	**Coefficient**	**p-value (Student’s t-test)**
Mean pressure (hPa)	− 0.0098	0.814
Absolute maximum daily temperature (°C)	0.2460	0.121
Daily mean temperature (°C)**	− 0.8448	**0.080**
Absolute minimum daily temperature (°C)	0.0005	0.997
Mean dew point (°C)	0.7026	0.127
Mean relative humidity (%)**	− 0.1921	**0.089**
Mean amount of cloud (%)	− 0.0029	0.695
Total rainfall (mm)	− 0.0105	0.447
Total bright sunshine (hours)*	− 0.1303	**0.031**
Mean wind speed (km/h)	0.0109	0.322
Prevailing wind direction (EW, °)*	0.0070	**0.011**
Prevailing wind direction (NS, °)	0.0019	0.514

Results of the overall model fit and parameter estimates are compiled to examine the weather influence on the bird activities. Hypothesis testings were carried out at a significant level of 0.05.

Bold values indicate the p-values less than 0.05 (statistically significant) and 0.10 (marginally significant).

*Individual weather factors that are statistically significant to the count of all birds (p-value < 0.05).

**Individual weather factors that are marginally significant to the count of all birds (p-value < 0.10).

## Discussion

Here we leverage domain randomization to enhance the accuracy of the bird detection models. We create synthetic data, i.e., virtual egrets merged on real background, to tackle the lack of labeled bird data. Importantly, we demonstrate that by pretraining deep learning models with synthetic data of sufficient variations, we can force the model to focus on the fine-grained features of the great egrets and little egrets, which are the crucial features used by human experts for bird detection. This could be useful and applicable for the detection of other bird species with limited labeled data, of which the features are highly similar (e.g., species under the same family).

We explore the application of domain randomization-enhanced deep learning models based on the 2-months monitoring data of one testing site. Our findings provided multiple potential advantages over conventional monitoring by the visual technique and human endurance. Our deep learning-based object detection enables extreme intensive surveys for bird count of different species, behavioural study, spatial preferences and inter- and intra-specific interaction, under different weather conditions (i.e., 12 weather factors were used in this study). In our study, for instance, an influx of the total bird counts might indicate the presence of migratory birds, which could be useful for the study of the bird migratory behaviour and pattern. This new technology could therefore be applied in important breeding, stopover or wintering sites, e.g. the RAMSAR wetlands or Important Bird and Biodiversity Areas (IBAs), in order to monitor their number, and arrival and departure times. A high speed and resolution camera could further allow assessment of their diets and the search time for prey items. Ornithologists could also examine the hotspots of the bird intensity maps and the influence of different weather factors (e.g., wind intensity, relative humidity, temperature) to investigate the nest site selection and habitat preferences of certain bird species across different periods, e.g., during the breeding season. Furthermore, the daily record of the bird departure and return times facilitates the planning of environmental monitoring and audit. Construction activities, which inevitably create noise, could be scheduled at periods with the least numbers of birds, thereby minimizing disturbance to birds during inhabiting, foraging and breeding activities. Following this study, the established framework can be applied to other sites for monitoring same/different bird species, so as to implement more thorough investigation to obtain more conclusive evidences in studying the bird behaviour. Furthermore, datasets of different bird species can be continuously collected and annotated to augment the size of training set. While automating the bird detection, more sophisticated deep learning models and training strategies can also be implemented to enhance the model accuracy. To summarise, this study presents a paradigm shift from the conventional bird detection methods. The domain randomization-enhanced deep learning detection models (together with the Green AI Camera) enables wide-scale, long-term and end-to-end bird monitoring, in turn formulating better habitat management, conservation policies and protective measures.

## Methods

### Data collection and annotation

We invented a tailor-made Green AI Camera (see Fig. 1a). The “Green” term refers to the application related to environmental monitoring and ecological conservation, and the “AI” term, that represents Artificial Intelligence, refers to the combination of different types of machine learning and deep learning algorithms used to analyse the huge amounts of data generated from this data collection system. Green AI camera exhibits several advantages to meet the needs of long-term monitoring at outdoor areas: (i) automatic continuous recording; (ii) high-resolution videos; (iii) high-frame rate videos; (iv) huge local data storage; and (v) protection over harsh environments (e.g., extreme weather conditions). As shown in Fig. 1a, six cameras of 4 K resolution are used to continuously render videos of wide field of view. The videos are acquired at a rate of 25 frames per second with High Efficiency Video Coding (also known as H.265) for preserving the visual quality of high-resolution inputs. The external part of the Camera is an optical window composed of two parallel curved surfaces, that provides protection to the electronic sensors and detectors, and at the same time, ensures clear and distortion free field of view.

In this project, we installed the Green AI Camera at Penfold Park, Hong Kong, China (22° 23′ 57.22″ N, 114° 12′ 24.42″ E; see Fig. 1b for the aerial view of the study site) to monitor the bird behaviour at trees in the centre of a pond (see Fig. 1c for the recorded background image). The dominant bird species found at this park are great egrets (*Egretta alba*) and little egrets (*Egretta garzetta*). Other birds includes black-crowned night herons (*Nycticorax nycticorax*), Chinese pond herons (*Ardeola bacchus*) and eastern cattle egrets (*Bubulcus ibis*)^7^. The data used in this study covered two periods, i.e., 2019-05-13–2019-09-13 and 2019-09-23–2019-11-26 (62 days); the recorded time for each day was from 04:00 to 20:00, as the usage of infrared is prohibited at the study site (which is completely dark at night). For the first period, we annotated 900 images from the data period of 2019-05-13–2019-06-08 for model training, 100 images from the data period of 2019-08-01–2019-08-17 for model validation, and 110 images from the data period of 2019-08-18–2019-09-13 for model testing. For the second period, this 2-months continuous monitoring data was used for the analyses of the egret behaviour, based on the detected egrets from the trained model. We annotated images by drawing bounding boxes for the observed birds; three labels, which were great egrets, little egrets and other birds (most of them were black-crowned night herons), were used, and the bird counts for each label were 2591, 1401 and 372. Our labels were sent for review by ornithologists for verification. The image dimension was 2139 × 1281 pixels; both length and width of the bounding boxes of birds were within the range of 42 and 160 pixels, and the average and maximum sizes of birds were ~ 0.095% and 2.94% of the image, respectively.

### Domain randomization

Domain randomization enables the generation of 3D models with desired features and rendering them on specific 2D backgrounds. We used the open-source 3D graphics toolset Blender (v2.79) to create virtual birds, i.e., a great egret and a little egret. Other birds, such as black-crowned night herons, were not considered as their features are highly distinctive compared to egrets. During the development of the virtual images, we applied a large variation of the prominent features (e.g., pose, and body size presented in images due to varying distanced from camera), and environmental and operational conditions (e.g., lighting condition, camera viewpoint and bird location in images), which in turn forced the models to focus on the fine-grained bird features. We used Inverse Kinematics to adjust the armature (bones) of the birds to create different poses. Then, we carefully selected background images that contained no real egrets for creating synthetic images. We merged the 3D models onto the 2D backgrounds, by pasting the virtual egrets using Gaussian paste, Poisson paste and direct paste, at any reasonable location of the images. When pasting, the bird size distribution was set as uniform, ranging from 0.04% to 0.56% of the background dimension. Other attributes included applying light at a uniform distribution between 0.6 and 1.0 of the maximum light values of Blender, and setting the camera viewpoint as a uniform joint distribution of the three Euler angles. All these computing procedures were deployed by creating a python plug-in for Blender. A total of 1000 synthetic images was created for model pretraining.

### Network architecture and training details

We selected Faster R-CNN^32^ (Fig. 3a) for performing object detection, due to its satisfactory performance in similar tasks^14^. Faster R-CNN is a two-stage detector. In the first stage, Faster R-CNN extracts feature maps from input image using a convolutional layer (see Fig. 3a of the manuscript) and proposes potential image regions that contain target objects with a regional proposal network. In the second stage, based on the proposed regions, Faster R-CNN extracts the bounding box and the category information of objects using a region of interest head. In this study, the term “convolutional layer” refers to a feature extraction backbone used to extract and learn features from inputs; a ResNet-50^45^, of which the residual networks have been useful, was used as the backbone in this study. Furthermore, we adopted Feature Pyramid Network^46^, which uses a top-down structure with lateral connections to produce feature maps at all scales, to fuse the multi-scale information^46^, so as to enhance the detection of tiny objects in small scale (birds only occupy small areas of the recorded images). The overall architecture of our model was similar to the RetinaNet^34^, except that the cross entropy was used as the loss function in our model, instead of the focal loss. The focal loss, which was used to tackle the problem of imbalanced datasets, was not considered as the usage of synthesized datasets has balanced the proportion of the great egrets and little egrets.

We applied stochastic gradient descent as the optimizer for model training, with a weight decay of 0.0001, a momentum of 0.9 and a learning rate of 0.0005. We first pretrained the Faster R-CNN with the synthetic images, then fine-tuned the pretrained model with the real images. The training was deployed with two GeForce RTX 2080 Ti GPUs and the batch size was two per GPU. For comparison, we used the attention mechanism^15^ to build similar object detection models (see the dotted region in Fig. 3b for the attention mechanism) under the same training settings.

### Model evaluation metrics

We adopted the commonly used mean average precision (mAP) to evaluate the model performance. The mAP metric jointly takes precision, recall and intersection over union (IoU) into consideration, where precision measures how relevant the predictions are based on all the retrieved instances; recall reflects the fractions of the relevant instances that are actually retrieved; and IoU defines the ratio intersection and union of the predicted and ground-truth bounding boxes. For a specific IoU, mAP is computed by averaging the precision value over the recall values from 0 to 1. For all analysed cases, we trained the model ten times and ran inference for all the individual trained models. The mAP (IoU = 0.5) was reported in the format of “mean ± standard deviation”. We also plotted the precision-recall curves (at IoU = 0.5) for all four cases.

### Perception made by models for bird detection

We attempted to visualize the feature maps produced by deep learning models, to shed light on how models localize and classify different bird species. However, noise is presented in the feature maps. Such noise effects are located at the null space of the matrix of the affine transformation operator following these feature maps in the network, and are set to zero vectors by the affine transformation and eventually omitted by the network. Therefore, in order to effectively visualize the feature maps without the noise influence, we split the features into row space and null space of the aforementioned matrix, followed by extracting the row-subspace features to visualize the model-related information^61^. Supposing that the matrix of the affine transformation operation is $A$, the feature maps are $x$, the coordinates of $x$ in the row subspace are $\widehat{x}$, the feature maps in the row subspace are ${x}_{r}$ and the feature maps in the null subspace are ${x}_{n}$, the decomposition could be performed by the following Eqs. ^61^:

1 $$A{A}^{T}\widehat{x}=Ax$$

2 $${x}_{r}={A}^{T}\widehat{x}$$

3 $${x}_{n}=x-{x}_{r}$$

After extracting the row-subspace features, the dimensions of the remaining feature maps were in the hundreds, which is difficult to visualize. Therefore, we applied principal component analysis (PCA) to reduce the dimensions of the remained feature maps to three and then used the weighted average of these three dimensions for visualization. As the first dimension usually carries the most important information, we applied the heaviest weight on the first dimension, followed by the second and third dimensions. The weights used herein were the coefficients used to convert RGB images to grayscale images:

4 $$V=0.7152{V}_{1}+0.2126{V}_{2}+0.0722{V}_{3}$$

where $V$ is the weighted feature map, and ${V}_{1}$, ${V}_{2}$ and ${V}_{3}$ are the first, second and third dimensions after applying PCA.

### Heatmap showing the spatial distribution of egrets

Heatmaps were created to visualize the spatial distribution of egrets based on the random field theory. We first partitioned the recorded video frames into cells of 200 × 200 pixels. For each grid, we applied a spatial smoothing to estimate the count of the ${k}^{th}$ bird species ($k=1$ for the great egret, $k=2$ for the little egret) at time $t$:

5 $${x}_{t,{p}_{i},k}={\sum }_{j=1}^{n}{e}^{-\upbeta d\left({p}_{i},{p}_{j}\right)}c\left(t,{p}_{i},k\right)$$

where ${x}_{t,{p}_{i},k}$ is the count of the ${k}^{th}$ bird species of cell ${p}_{i}$ at time $t$, after spatial smoothing over all $n$ cells; $d\left({p}_{i},{p}_{j}\right)$ is the Euclidean distance between the central points of the cells ${p}_{i},{p}_{j}$; $c\left(t,{p}_{i},k\right)$ is the number of birds located at the corresponding cell; and $\upbeta$ is a smoothing constant, satisfying $\upbeta \ge 0$. Following that, we applied an exponential smoothing on ${x}_{t,{p}_{i},k}$:

6 $${s}_{t,{p}_{i},k}=\uplambda {x}_{t,{p}_{i},k}+\left(1-\uplambda \right){s}_{t-1,{p}_{i},k}$$

where ${s}_{t,{p}_{i},k}$ is the count of the ${k}^{th}$ bird species of cell ${p}_{i}$ at time $t$ after spatial and temporal smoothing; and $\mathrm{\alpha }$ is a smoothing constant, with $0\le\uplambda \le 1$. After computing all ${s}_{t,{p}_{i},k}$, we averaged them over time to create the heatmaps within a specified period.

### Statistical analyses

We computed the Pearson correlation coefficient to identify the correlation between the counts of the great egrets and little egrets. The daily schedule of the great egrets and little egrets were studied with two hypothesis testings using the following null hypotheses: (i) the departure time of the great egrets and little egrets are same (HT1, tested with a two-tailed Student’s t-test); and (ii) the return time of the great egrets is equal to or later than the little egrets (HT2, tested with a one-tailed Student’s t-test). A significant level of 0.05 was chosen for all hypothesis testings. We also built a multivariable linear regression model to study the weather influence on bird activities. Prior to that, hypothesis testing (HT3) was conducted with a two-tailed Student’s t-test to examine whether data detrending was required, by stating a null hypothesis of “the time does not have influence on the bird count-weather relationship”. Detrending was conducted to eliminate the time factor that might bias the bird counts:

7 $${\mathrm{ln}}\left({\ddot{y}}_{t}\right)={\mathrm{ln}}\left({y}_{t}\right)-{\widehat{\alpha }}_{0}-{\widehat{\alpha }}_{1}t-{\widehat{\alpha }}_{2}{t}^{2}$$

8 $${\ddot{x}}_{ti}={x}_{ti}-{\widehat{\gamma }}_{0i}-{\widehat{\gamma }}_{1i}t-{\widehat{\gamma }}_{2i}{t}^{2}$$

where ${y}_{t}$ and ${\ddot{y}}_{t}$ are respectively the original and detrended bird counts at the time step $t$; ${x}_{ti}$ and ${\ddot{x}}_{ti}$ are respectively the original and detrended *i*^th^ weather factor at $t$, and ${\widehat{\alpha }}_{0}$, ${\widehat{\alpha }}_{1}$, ${\widehat{\alpha }}_{2}$, ${\widehat{\gamma }}_{0i}$, ${\widehat{\gamma }}_{1i}$ and ${\widehat{\gamma }}_{2i}$ are the regression coefficients. The multivariable linear regression model was then built with detrended ${\ddot{y}}_{t}$ and ${\ddot{x}}_{ti}$:

9 $${\mathrm{ln}}\left({\widehat{\ddot{y}}}_{t} \right)=\sum_{i=1}^{n}{\widehat{\beta }}_{i}{\ddot{x}}_{ti}$$

where ${\widehat{\ddot{y}}}_{t}$ is the fitted bird counts at time step $t$, $n$ is the total number of weather factors and ${\widehat{\beta }}_{i}$ is the regression coefficient.

## Supplementary Information

Supplementary Information

## References

[1] Yong W, Finch DM, Moore FR, Kelly JF (1998). Stopover ecology and habitat use of migratory Wilson’s warblers. Auk.

[2] Cherry JD (1982). Fat deposition and length of stopover of migrant white-crowned sparrows. Auk.

[3] Woodrey MS, Moore FR (1997). Age-related differences in the stopover of fall landbird migrants on the Coast of Alabama. Auk.

[4] Murphy-Klassen HM, Underwood TJ, Sealy SG, Czyrnyj AA (2005). Long-term trends in spring arrival dates of migrant birds at delta marsh, Manitoba, relation to climate change. Auk.

[5] Bollinger EK (1995). Successional changes and habitat selection in hayfield bird communities. Auk.

[6] Marzluff JM, Knick ST, Vekasy MS, Schueck LS (1997). Spatial use and habitat selection of golden eagles in Southwestern Idaho. Auk.

[7] Anon. Summer 2018 Report: Egretry Counts in Hong Kong with particular reference to the Mai Po Inner Deep Bay Ramsar Site. *Hong Kong Bird Watch. Soc. Agric. Fish. Conserv. Dep. Hong Kong Spec. Adm. Reg. Gov.* (2018).

[8] Sutter, E. *Radar als Hilfsmittel der Vogelzugsforschung*. (Verlag Nicht Ermittelbar, 1957).

[9] Lack D, Varley G (1945). Detection of birds by radar. Nature.

[10] Abd-Elrahman A, Pearlstine L, Percival F (2005). Development of pattern recognition algorithm for automatic bird detection from unmanned aerial vehicle imagery. Surv. Land Inf. Sci..

[11] Wu T, Luo X, Xu Q (2018). A new skeleton based flying bird detection method for low-altitude air traffic management. Chin. J. Aeronaut..

[12] T’Jampens, R., Hernandez, F., Vandecasteele, F. & Verstockt, S. Automatic detection, tracking and counting of birds in marine video content. in *2016 Sixth International Conference on Image Processing Theory, Tools and Applications (IPTA)* 1–6 (2016).

[13] May R, Steinheim Y, Kvaløy P, Vang R, Hanssen F (2017). Performance test and verification of an off-the-shelf automated avian radar tracking system. Ecol. Evol..

[14] Hong S-J, Han Y, Kim S-Y, Lee A-Y, Kim G (2019). Application of deep-learning methods to bird detection using unmanned aerial vehicle imagery. Sensors.

[15] Hu, T. & Qi, H. See Better Before Looking Closer: Weakly Supervised Data Augmentation Network for Fine-Grained Visual Classification. Preprint at https://arxiv.org/abs/1901.09891 (2019).

[16] Wen B, Li K, Zhang Y, Zhang B (2020). Cancer neoantigen prioritization through sensitive and reliable proteogenomics analysis. Nat. Commun..

[17] Zheng X (2020). Deep learning radiomics can predict axillary lymph node status in early-stage breast cancer. Nat. Commun..

[18] Dwivedi SK, Tjärnberg A, Tegnér J, Gustafsson M (2020). Deriving disease modules from the compressed transcriptional space embedded in a deep autoencoder. Nat. Commun..

[19] Golestani N, Moghaddam M (2020). Human activity recognition using magnetic induction-based motion signals and deep recurrent neural networks. Nat. Commun..

[20] Wu S (2019). Artificial intelligence reveals environmental constraints on colour diversity in insects. Nat. Commun..

[21] Park S, Kwak W, Lee HK (2020). Accelerated spin dynamics using deep learning corrections. Sci. Rep..

[22] Eun D (2020). Deep-learning-based image quality enhancement of compressed sensing magnetic resonance imaging of vessel wall: Comparison of self-supervised and unsupervised approaches. Sci. Rep..

[23] Lee C (2020). Classification of femur fracture in pelvic X-ray images using meta-learned deep neural network. Sci. Rep..

[24] Adhikari B (2020). A fully open-source framework for deep learning protein real-valued distances. Sci. Rep..

[25] Zou, Z., Shi, Z., Guo, Y. & Ye, J. Object Detection in 20 Years: A Survey. Preprint at https://arxiv.org/abs/1905.05055 (2019).

[26] Jiao L (2019). A survey of deep learning-based object detection. IEEE Access.

[27] He, K., Girshick, R. & Dollár, P. Rethinking Imagenet Pre-training. in *Proceedings of the IEEE International Conference on Computer Vision* 4918–4927 (2019).

[28] Szegedy, C. *et al.* Going Deeper with Convolutions. in *Proceedings of the IEEE conference on computer vision and pattern recognition* 1–9 (2015).

[29] Yoshihashi, R., Kawakami, R., Iida, M. & Naemura, T. Evaluation of Bird Detection using Time-Lapse Images Around a Wind Farm. in *European Wind Energy Association Conference* (2015).

[30] Takeki, A. *et al.* Detection of Small Birds in Large Images by Combining a Deep Detector with Semantic Segmentation. in *2016 IEEE International Conference on Image Processing (ICIP)* 3977–3981 (2016).

[31] Takeki A (2016). Combining deep features for object detection at various scales: finding small birds in landscape images. IPSJ Trans. Comput. Vis. Appl..

[32] Ren S, He K, Girshick R, Sun J (2017). Faster R-CNN: Towards real-time object detection with region proposal networks. IEEE Trans. Pattern Anal. Mach. Intell..

[33] Redmon, J., Divvala, S., Girshick, R. & Farhadi, A. You Only Look Once: Unified, Real-time Object Detection. in *Proceedings of the IEEE conference on computer vision and pattern recognition* 779–788 (2016).

[34] Lin, T.-Y., Goyal, P., Girshick, R. B., He, K. & Dollár, P. Focal loss for dense object detection. Preprint at https://arxiv.org/abs/1708.02002 (2017). 10.1109/TPAMI.2018.2858826 30040631

[35] LeCun Y, Bottou L, Bengio Y, Haffner P (1998). Gradient-based learning applied to document recognition. Proc. IEEE.

[36] Lin, T.-Y., RoyChowdhury, A. & Maji, S. Bilinear CNN Models for Fine-grained Visual Recognition. in *2015 IEEE International Conference on Computer Vision (ICCV)* 1449–1457 (2015).

[37] Dai X, Gong S, Zhong S, Bao Z (2019). Bilinear CNN model for fine-grained classification based on subcategory-similarity measurement. Appl. Sci..

[38] Ge, W., Lin, X. & Yu, Y. Weakly Supervised Complementary Parts Models for Fine-Grained Image Classification from the Bottom Up. in *Proceedings of the IEEE Conference on Computer Vision and Pattern Recognition* 3034–3043 (2019).

[39] Cui, Y., Song, Y., Sun, C., Howard, A. & Belongie, S. Large Scale Fine-grained Categorization and Domain-Specific Transfer Learning. in *Proceedings of the 2018 IEEE/CVF Conference on Computer Vision and Pattern Recognition* 4109–4118 (2018).

[40] Ngiam, J. *et al.* Domain Adaptive Transfer Learning with Specialist Models. Preprint at https://arxiv.org/abs/1811.07056 (2018).

[41] Kang B, Lee Y (2020). High-resolution neural network for driver visual attention prediction. Sensors.

[42] Peng, X. B., Andrychowicz, M., Zaremba, W. & Abbeel, P. Sim-to-Real Transfer of Robotic Control with Dynamics Randomization. *2018 IEEE Int. Conf. Robot. Autom. ICRA* 3803–3810 (2018).

[43] Tobin, J. *et al.* Domain randomization for transferring deep neural networks from simulation to the real world. Preprint at https://arxiv.org/abs/1703.06907 (2017).

[44] Sadeghi, F. & Levine, S. Cad2rl: Real single-image flight without a single real image. Preprint at https://arxiv.org/abs/1611.04201 (2016).

[45] He, K., Zhang, X., Ren, S. & Sun, J. Deep residual learning for image recognition. in *Proceedings of the 2016 IEEE Conference on Computer Vision and Pattern Recognition* 770–778 (2016).

[46] Lin, T.-Y. *et al.* Feature pyramid networks for object detection. in *Proceedings of the IEEE conference on computer vision and pattern recognition* 2117–2125 (2017).

[47] Carey G (2001). The Avifauna of Hong Kong.

[48] HKBWS. The First GPS-Tracking Research of Egrets in Hong Kong Fishpond Plays A Key Role for Both Resident and Migratory Little Egret. *The Hong Kong Bird Watching Society* https://cms.hkbws.org.hk/cms/en/hkbws/egret-tracking-2 (2018).

[49] Young L (1998). The importance to ardeids of the deep bay fish ponds, Hong Kong. Biol. Conserv..

[50] Choi Y-S, Kwon I-K, Yoo J-C (2007). Foraging habitat preferences of herons and egrets. J. Ecol. Environ..

[51] Pang RH, Yu TL, Busam M (2019). Low breeding success of the little egret (Egretta garzetta) near residential areas and in colonies exposed to gales: A comparison of colony in Sichuan, Southwest China, with literature. Anim. Cells Syst..

[52] Post W (1990). Nest survival in a large ibis-heron colony during a three-year decline to extinction. Colon. Waterbirds.

[53] Hilaluddin J, Shah N, Shawl T (2003). Nest site selection and breeding success by cattle egret and little egret in Amroha, Uttar Pradesh, India. Waterbirds.

[54] HKO. Hong Kong Observatory Open Data. *Hong Kong Observatory* https://www.hko.gov.hk/en/cis/climat.htm (2019).

[55] Wiese JH (1976). Courtship and pair formation in the great egret. Auk.

[56] Moore FR (1987). Sunset and the orientation behaviour of migrating birds. Biol. Rev..

[57] Recher HF, Holmes RT, Davis WE, Morton S (1983). Foraging behavior of Australian herons. Colon. Waterbirds.

[58] Pinto, D., Chivittz, C., Bergmann, F. & Tozetti, A. Microhabitat use by three species of egret (Pelecaniformes, Ardeidae) in southern Brazil. *Braz. J. Biol.* **73**, 791–796 (2013). 10.1590/s1519-69842013000400015 24789395

[59] Corrêa TC, Del Lama SN, De Souza JR, Miño CI (2016). Genetic structuring among populations of the Great Egret, Ardea alba Egretta, in major Brazilian wetlands: Genetic structuring in great egret populations. Aquat. Conserv. Mar. Freshw. Ecosyst..

[60] Smith, J. P. An energy-circuit population model for Great Egrets (Ardea alba) at Lake Okeechobee, Florida, USA. *Ecol. Model.* **97**, 1–21 (1997).

[61] Mao, X., Su, Z., Tan, P. S., Chow, J. K. & Wang, Y.-H. Is Discriminator a Good Feature Extractor? Preprint at https://arxiv.org/abs/1912.00789 (2019).

