# Supplementary material for: Domain randomization-enhanced deep learning models for bird detection
Source: Sci Rep. 2021 Jan 12;11:639. doi: 10.1038/s41598-020-80101-x (PMC7803967; doi:10.1038/s41598-020-80101-x)
Supplement: Supplementary file 1 — Supplementary Information [file 41598_2020_80101_MOESM1_ESM.pdf]

Manuscript title: Domain randomization-enhanced deep learning models for bird detection

Authors:

Xin MAO, Jun Kang CHOW, Pin Siang TAN, Kuan-fu LIU, Jimmy WU, Zhaoyu SU, Ye Hur CHEONG, Ghee Leng OOI, Chun Chiu PANG, Yu-Hsing WANG*

*Corresponding author

Supplementary Video:

The authors sincerely apologise for not directly uploading the analysed video upon the submission of manuscript, as the video size (~1.7 Gb) exceeds the allowed file size.

Please kindly refer the link below for the analysed video of egrets staying at Penfold Park, Hong Kong, that covers the monitoring period of 2019-09-23 – 2019-11-26:

Link for the full-length video:

Supplementary_Video_Bird_detection

Link for the compressed version:

Supplementary_Video_Bird_detection_compressed

Other information:

The domain randomization-enhanced model achieves a mean average precision of 87.65%, based on the test set (the video recorded during the data period of 2019-08-18 – 2019-09-13). Future works can be implemented to continuously augment the model performance in bird detection. For instance, datasets of different bird species can be continuously collected and annotated to augment the size of training set. While automating the bird detection, more sophisticated deep learning models and training strategies can also be implemented to enhance the model accuracy.
